# Supplementary material for: Mechanisms for destabilisation of RNA viruses at air-water and liquid-liquid interfaces
Source: Nat Commun. 2021 Nov 24;12:6812. doi: 10.1038/s41467-021-27052-7 (PMC8613244; doi:10.1038/s41467-021-27052-7)
Supplement: Supplementary file 1 — Supplementary Information [file 41467_2021_27052_MOESM1_ESM.pdf]

Mechanisms for destabilisation of RNA viruses at air-water and liquid-liquid interfaces: Supplementary Information

C. A. Brackley, A. Lips, A. Morozov, W. C. K. Poon, D. Marenduzzo

Supplementary Figures

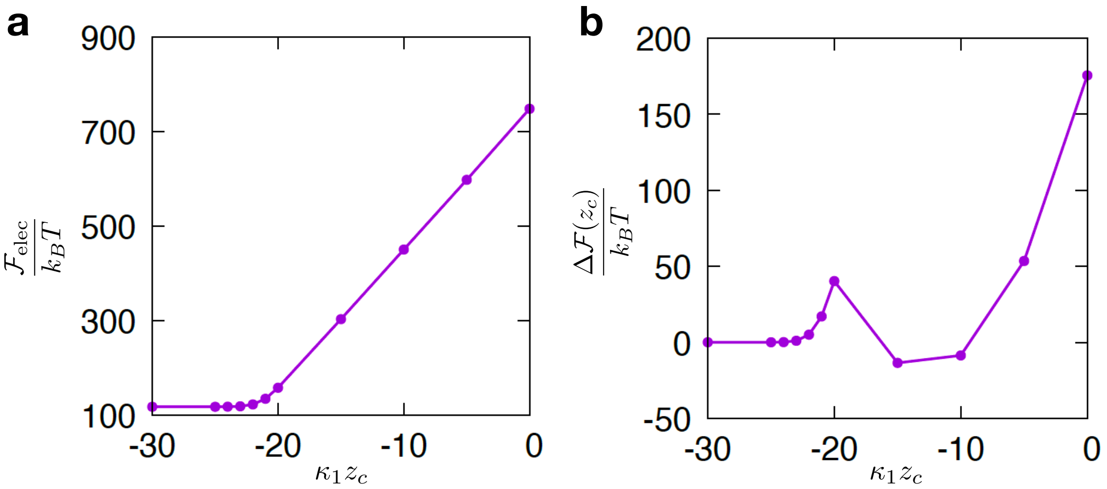

Supplementary Figure 1: **Free energy for an RNA virus approaching an ethanol-water interface.** (a) Approach curve showing the increase in electrostatic free energy (or self-energy) $\mathcal{F}_{\mathrm{elec}}(z_c)$ as a function of distance to the interface, for two media with $\epsilon_2/\epsilon_1 = 0.3125$ and $\kappa_2/\kappa_1 = 0.05$. This is a typical situation for an interface between two media with different and finite Debye lengths (which is different to the case of an air-water interface where $\kappa_2 = 0$); the parameter values are relevant for an ethanol-water interface (see text). (b) Plot of the total (electrostatic plus Pickering) free energy change, $\Delta\mathcal{F}(\kappa_1 z_c) = \mathcal{F}(\kappa_1 z_c) - \mathcal{F}(\kappa_1 z_c \to -\infty)$, as a virion approaches the interface modelled in (a) (assuming a surface tension of $\gamma = 1.5$ mN/m as in Fig. 3 of the main text).

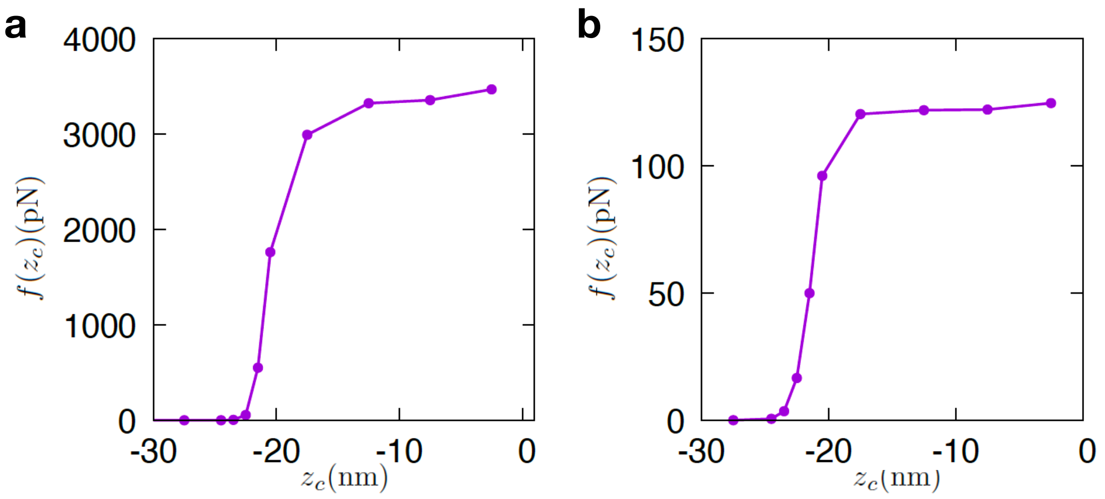

Supplementary Figure 2: **Electrostatic force curves.** Plot of the electrostatic force $f(z_c) = -\frac{\partial \mathcal{F}_{\rm elec}}{\partial z_c}$, felt by a virion as a function of distance to the interface, z_c, for (a) an air-water interface (parameters as in Fig. 2 of the main text) and (b) an ethanol-water interface (parameters as in Fig. S1). To express the value of the force in pN, we considered $\kappa_1^{-1} = 1$ nm.

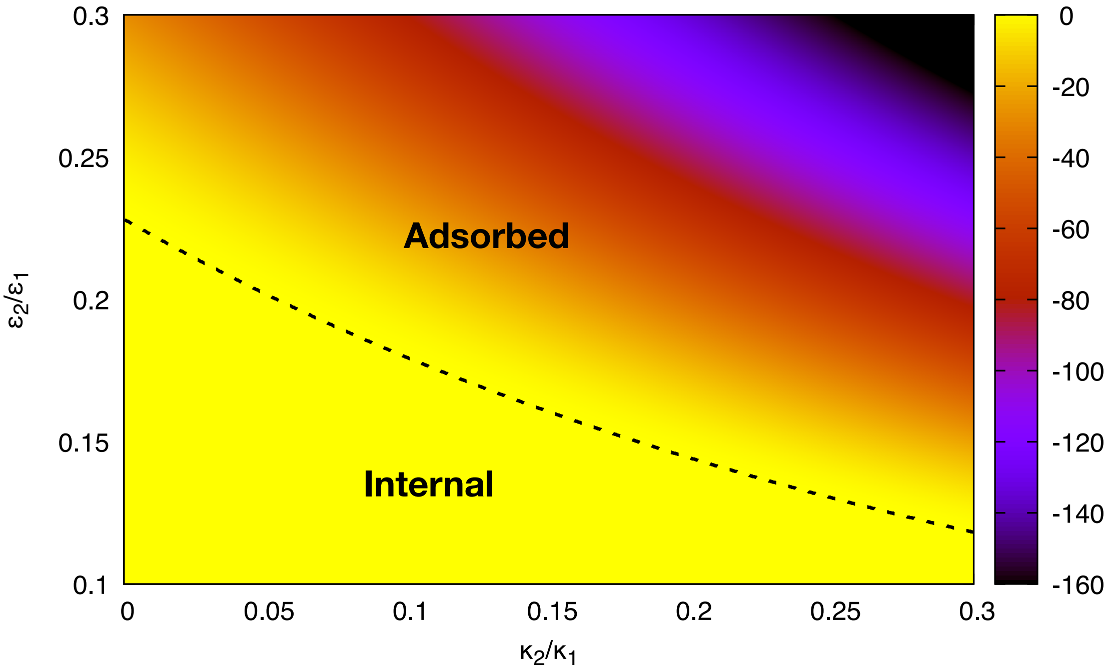

Supplementary Figure 3: **Phase diagram found by our scaling theory.** Phase diagram showing the fate of a viral particle approaching an interface between a physiological aqueous medium and another medium with variable electrostatic parameters, found by using our scaling theory. The heatmap shows the value of the adsorption free energy, Eq. (S26), in units of $k_B T$. The dashed line shows the line where the adsorption free energy is zero (equivalently $z_c^* = -R$). The phase diagram in the Figure corresponds to $\sigma^* = 11$, $\kappa_1 = 1$ nm^{-1}, $\delta = 2.5$ nm, $\epsilon_1 = 80\epsilon_0$, $\gamma = 1.5$ mN/m.

Supplementary Note 1: Poisson-Boltzmann numerics

In the main text we show numerical solutions of the non-linear Poisson-Boltzmann (PB) equation for an RNA virion close to an interface between two media, characterised by different values of the dielectric constant and Debye length. The problem has cylindrical symmetry, and we call z the position along the axis perpendicular to the interface ($z = 0$ denotes the interface plane), and r the distance to the centre of the viral particles in the plane parallel to the interface. In this geometry, the PB equation for monovalent salt electrolytes is,

$$\nabla \cdot \left(\epsilon(r,z)\nabla\tilde{\phi}\right) - \epsilon(r,z)\kappa^2(r,z)\sinh(\tilde{\phi}) = -\tilde{\rho}(r,z) \tag{S1}$$

where $\tilde{\phi} \equiv \frac{e_0\phi}{k_BT}$ is the dimensionless electrostatic potential, and $\tilde{\rho} = \frac{e_0\rho}{k_BT}$. To model an RNA virus, we considered a charge distribution $\tilde{\rho(r,z)}$ consisting of two concentric charged shells: an interior negatively charged shell (representing RNA) and an exterior positively charged one (representing the viral capsid, see Fig. 1 in the main text). The explicit functional form for $\tilde{\rho}(r,z)$ we considered is

$$\tilde{\rho}(r,z) = \rho_0\left[e^{-\alpha(\tilde{r}-R-\delta)^2} - e^{-\alpha(\tilde{r}-R+\delta)^2}\right], \tag{S2}$$

where $\tilde{r} = \sqrt{r^2 + (z - z_c)^2}$, R is the capsid radius, 2δ is the distance between the two charged shells, while α controls the width of each shell. As mentioned in the main text, ϵ and κ vary in medium I, II, and III (the capsid interior, see Fig. 1 in the main text); their values are respectively called ϵ_1 and κ_1 (in medium I), ϵ_2 and κ_2 (in medium II), ϵ_3 and κ_3 (in medium III). In our numerical calculations we chose $\epsilon_1 = 80\epsilon_0$ (with ϵ_0 the dielectric permittivity of vacuum) and $\kappa_1 = 1$ nm^{-1} to model the aqueous phase containing the virion, $\epsilon_3 = \epsilon_1$, and $\kappa_3 = \kappa_1$ to model the virus interior (which we assumed to be the same as medium I), whereas we varied κ_2 and ϵ_2. The case of an air-water interface corresponds to $\kappa_2 = 0$ and $\epsilon_2 = \epsilon_0$ (Fig. 2 of the main text), whereas in Fig. 3 of the main text we varied κ_2 between 0 and $0.3\kappa_1$ and ϵ_2 between $0.1\epsilon_1$ and $0.3\epsilon_1$ to model a liquid-liquid interface. The self-energy as a function of distance to the interface for a liquid-liquid interface with $\epsilon_2/\epsilon_1 = 0.3125$ and $\kappa_2/\kappa_1 = 0.05$ is shown in Fig. 1, together with the total change in free energy (including the Pickering contribution): this case is relevant for the situation where medium II is an alcohol rub rich in ethanol (we refer to the corresponding interface as an ethanol-water interface for simplicity). The electrostatic forces opposing adsorption, for both an air-water and an ethanol-water interface, are given in Suppl. Fig. 2.

In our numerical calculations we set $\kappa_1 = \epsilon_1 = 1$, $R = 20$, $\delta = 2.5$, $\rho_0 = 0.5$, $\alpha = 1$. Because ϕ is given in units of k_BT/e_0 as the potential in Eq. (S1) has been made dimensionless, it follows that charge densities in Eq. (S1) are measured in units of $k_BT\epsilon_1\kappa_1^2/e_0$. With these choices, a free energy simulation unit equals $(k_BT)^2\epsilon_1/(e_0^2\kappa_1)$.

To solve Eq. (S1), we used a finite difference scheme in cylindrical coordinates, with a 400×600 grid, and a spatial discretisation of $\Delta x = 0.5$ along both r and z. We used a relaxation algorithm, introducing a time derivative so that Eq. (S1) is solved in steady state. The time step in the relaxation algorithm was chosen to be $\Delta t = 0.15 \Delta x^2$, which is small enough for the algorithm to converge, yet large enough to not compromise computational efficiency.

To compute the electrostatic self free energy of the system, we start from the observation that it can be written as

$$\mathcal{F}_{\text{elec}}(z_c) = \int d\mathbf{r} \left[-\frac{\epsilon}{2} \left(\nabla\phi\right)^2 + \rho\phi + \epsilon\kappa^2 \left(\frac{k_B T}{e_0}\right)^2 \left(1 - \cosh(\tilde{\phi})\right) \right], \tag{S3}$$

where $\int d\mathbf{r}$ denotes integration over the whole simulation domain. That this formula is the appropriate one can be shown by noting that when minimising Eq. (S3) with respect to ϕ – i.e., when setting the functional derivative of Eq. (S3) equal to 0 – we obtain Eq. (S1). Note that we have added a term independent of ϕ to $\mathcal{F}_{\text{elec}}(z_c)$ such that this quantity is 0 when $\phi \equiv 0$. If we integrate by part the $-\frac{\epsilon}{2} \left(\nabla\phi\right)^2$ term in the integral in Eq. (S3), neglect the surface contribution (as $\phi = 0$ at the boundaries) and use Eq. (S1), we find that the electrostatic self energy can also be written as follows,

$$\mathcal{F}_{\text{elec}}(z_c) = \int d\mathbf{r} \left[\frac{1}{2}\rho\phi + \epsilon\kappa^2 \left(\frac{k_B T}{e_0}\right)^2 \left(\frac{\tilde{\phi}\sinh(\tilde{\phi})}{2} - \cosh(\tilde{\phi}) + 1 \right) \right]. \tag{S4}$$

Eq. (S4) is equal to Eq. (2) in the main text. Note that in the Debye-Hückel linearised approximation, the electrostatic free energy is simply given by

$$\mathcal{F}_{\text{elec}}(z_c) = \frac{1}{2} \int d\mathbf{r}\rho\phi. \tag{S5}$$

Supplementary Note 2: Self-energy calculations

We now analyse the idealised case of systems made up by infinitesimally thin shells, and compute their self-energy in the Debye-Hückel approximation, which provides a useful baseline framework to interpret the results found by simulating the non-linear Poisson-Boltzmann equation.

Self-energies of single-shell systems

It is useful to start with a single-shell system, which models an empty viral capsid. For a single shell of radius R, infinitesimal thickness, and surface charge density σ, the three-dimensional charge density entering the Poisson-Boltzmann equation can be simply written as

$$\rho(r) = \sigma\delta(r - R), \tag{S6}$$

where δ here denotes the Dirac delta function.

Let us first analyse the case of $\kappa = 0$ (no screening), which can be done by using standard electrostatics. We call ϵ the dielectric constant of the medium. In this case, the potential for $r \leq R$ needs to be a constant, as the electric field is 0 – since there are no charges inside the shell. For $r > R$, the potential must be the same of that of a point charge with charge $Q = 4\pi\sigma R^2$ (the total charge of the shell). Imposing continuity at $r = R$, we fix the constant and obtain that the electrostatic potential is given by

$$\begin{aligned} \phi(r) &= \frac{Q}{4\pi\epsilon R} \qquad r \leq R \\ \phi(r) &= \frac{Q}{4\pi\epsilon r} \qquad r > R. \end{aligned} \tag{S7}$$

As a consequence the self-energy for $\kappa = 0$ is

$$\mathcal{F}_{\text{elec}} = \frac{1}{2} Q\phi(R) = \frac{2\pi\sigma^2 R^3}{\epsilon}. \tag{S8}$$

Therefore for $\kappa = 0$ the self-energy of a single shell scales as R^3.

Let us now turn to the case of a shell in a medium where there are mobile charges and electrostatic screening, $\kappa \neq 0$. One way to obtain the electrostatic potential in the Debye-Hückel approximation is to solve the linearised Poisson-Boltzmann equation for a charged shell,

$$\nabla^2\phi - \kappa^2\phi = -\sigma\delta(r - R)/\epsilon. \tag{S9}$$

The general solution of Eq. (S9) for $r \neq R$ is

$$\phi(r) = A\frac{\cosh(\kappa r)}{r} + B\frac{\sinh(\kappa r)}{r} = \frac{A - B}{2}\frac{e^{-\kappa r}}{r} + \frac{A + B}{2}\frac{e^{\kappa r}}{r} \tag{S10}$$

For $r < R$, we need $A = 0$ for the solution to be well behaved as $r \to 0$ (where there is no singularity, as there is no point charge, or δ function in the charge density). For $r > R$, we need $A + B = 0$ as the potential needs to go to 0 as $r \to \infty$. Integrating Eq. (S9) over a thin shell around $r = R$ gives the discontinuity in $d\phi/dr$ (which is minus the electric field) at $r = R$ as

$$\left(\frac{d\phi}{dr}\right)_{r\to R^+} - \left(\frac{d\phi}{dr}\right)_{r\to R^-} = -\frac{Q}{4\pi\epsilon R^2}. \tag{S11}$$

This condition together with continuity at $r = R$ fixes the values of the constants so that the potential for $r \geq R$ is given by

$$\phi(r) = \frac{Q\sinh(\kappa R)e^{-kr}}{4\pi\epsilon\kappa R r}, \tag{S12}$$

which may be viewed as the screened potential of a single point particle with charge $Q\sinh(\kappa R)/(\kappa R)$. The self-energy of the system is

$$\mathcal{F}_{\text{elec}} = \frac{1}{2} Q\phi(R) = \frac{2\pi\sigma^2 R^2}{\epsilon\kappa(1 + \text{cotanh}(\kappa R))}, \tag{S13}$$

as found in [1, 2]. For $\kappa R \gg 1$, which is relevant for viral capsid shells, the scaling therefore changes from $\sim R^3$ to $\sim R^2/\kappa$.

Self-energies of concentric shell systems

In our cases the RNA virion is approximated by two concentric shells, of radius $R-\delta$ and $R+\delta$, and with charges $-\sigma$ and σ, representing the RNA and viral capsid respectively. Now the three-dimensional charge density entering the Poisson-Boltzmann equation is

$$\rho(r) = \sigma\left[\delta(r-R-\delta) - \delta(r-R+\delta)\right]. \tag{S14}$$

We call $Q_1 = 4\pi\sigma R_1^2$ and $Q_2 = 4\pi\sigma R_2^2$ the total charge of the inner and outer shell respectively.

Again, we consider the cases with $\kappa = 0$ and $\kappa \neq 0$ separately, in analogy with the single-shell section.

For $\kappa = 0$, reasoning analogous to that in the previous section leads to the following ansatz for ϕ,

$$\begin{aligned} \phi(r) &= A & r &\leq R_1 \\ \phi(r) &= B + C/r & R_1 &< r \leq R_2 \\ \phi(r) &= D/r & r &> R_2, \end{aligned} \tag{S15}$$

with A, B, C and D constants to be determined. Due to Gauss law (equivalently, by integrating the Poisson equation over spheres with radius just above R_1 and just above R_2), we find $C = \frac{Q_1}{4\pi\epsilon}$ and $D = \frac{Q_2 - Q_1}{4\pi\epsilon}$. Requiring continuity at $r = R_1$ and $r = R_2$ fixes the remaining constants, yielding the following solution

$$\begin{aligned} \phi(r) &= \frac{Q_2}{4\pi\epsilon R_2} - \frac{Q_1}{4\pi\epsilon R_1} & r &\leq R_1 \\ \phi(r) &= -\frac{Q_1}{4\pi\epsilon r} + \frac{Q_2}{4\pi\epsilon R_2} & R_1 &< r \leq R_2 \\ \phi(r) &= \frac{Q_2 - Q_1}{4\pi\epsilon r} & r &> R_2. \end{aligned} \tag{S16}$$

The self-energy of two concentric shells with opposite surface charge density is therefore given by:

$$\begin{aligned} \mathcal{F}_{\text{elec}} &= \frac{1}{2}\left[-Q_1\phi(R_1) + Q_2\phi(R_2)\right] \\ &= \frac{Q_1}{8\pi\epsilon}\left(\frac{Q_1}{R_1} - \frac{Q_2}{R_2}\right) + \frac{Q_2(Q_2 - Q_1)}{8\pi\epsilon R_2} \\ &= \frac{4\pi\delta\sigma^2}{\epsilon}\left[-(R-\delta)^2 + 2R(R+\delta)\right]. \end{aligned} \tag{S17}$$

From the last line of Eq. (S17), it can be seen that, if $\delta \ll R$, then the self-energy of the two-shell system scales as $\sim R^2\delta$, which is different from the $\sim R^3$ scaling of the single-shell system found previously. The same calculations can be repeated for arbitrary charges in the two shells, and it is interesting that in the limit of $\delta \ll R$ the scaling remains the same if the charge densities in the two shells are tuned such that $Q_1 = Q_2$ – in this case the expression for the self-energy simplifies as the second term in the second line of Eq. (S17) vanishes.

Let us now consider the case with screening, $\kappa \neq 0$. In each of the three regions $r < R_1$, $R_1 < r < R_2$, $r > R_2$, the general solution is of the form given in Eq. (S10). As in the single shell calculation, the solution is proportional to $\frac{\sinh(\kappa r)}{r}$ for $r < R_1$ and to $\frac{e^{-\kappa r}}{r}$ for $r > R_2$, due to the boundary conditions at $r = 0$ and $r \to \infty$. By integrating over infinitesimally small shells around $r = R_1$ and $r = R_2$ we obtain the conditions for the discontinuities of $\frac{d\phi}{dr}$,

$$\begin{aligned}\left(\frac{d\phi}{dr}\right)_{r\to R_1^+} - \left(\frac{d\phi}{dr}\right)_{r\to R_1^-} &= \frac{Q_1}{4\pi\epsilon R_1^2} \\ \left(\frac{d\phi}{dr}\right)_{r\to R_2^+} - \left(\frac{d\phi}{dr}\right)_{r\to R_2^-} &= -\frac{Q_2}{4\pi\epsilon R_2^2}.\end{aligned} \tag{S18}$$

Together with the continuity of ϕ at $r = R_1$ and $r = R_2$, these two conditions are sufficient to fix all remaining constants. The resulting potential for the two-shell system in the screened medium is

$$\begin{aligned}\phi(r) &= \left(\frac{Q_2 e^{-\kappa R_2}}{4\pi\epsilon\kappa R_2} - \frac{Q_1 e^{-\kappa R_1}}{4\pi\kappa\epsilon R_1}\right)\frac{\sinh(\kappa r)}{r} & r \le R_1 \\ \phi(r) &= \left[\frac{Q_2 e^{-\kappa R_2}}{4\pi\epsilon\kappa R_2} + \frac{Q_1 \sinh(\kappa R_1)}{4\pi\epsilon\kappa R_1}\right]\frac{\sinh(\kappa r)}{r} \\ &\quad - \frac{Q_1 \sinh(\kappa R_1)}{4\pi\epsilon\kappa R_1}\frac{\cosh(\kappa r)}{r} & R_1 < r \le R_2 \\ \phi(r) &= \left[\frac{Q_2 \sinh(\kappa R_2)}{4\pi\epsilon\kappa R_2} - \frac{Q_1 \sinh(\kappa R_1)}{4\pi\epsilon\kappa R_1}\right]\frac{e^{-\kappa r}}{r} & r > R_2.\end{aligned} \tag{S19}$$

It is interesting to note that the potential for $r > R_2$ is non-zero even if $Q_1 = Q_2$ in a medium with screening (i.e. $\kappa \neq 0$). In other words, the effective charge is a non-trivial combination of bare charges, screening length and geometric parameters. From Eq. (S19), the self-energy is found to be

$$\begin{aligned}\mathcal{F}_{\text{elec}} &= \frac{Q_1^2 e^{-\kappa R_1}\sinh(\kappa R_1)}{8\pi\epsilon\kappa R_1^2} + \frac{Q_2^2 e^{-\kappa R_2}\sinh(\kappa R_2)}{8\pi\epsilon\kappa R_2^2} \\ &\quad - \frac{Q_1 Q_2 e^{-\kappa R_2}\sinh(\kappa R_1)}{4\pi\epsilon\kappa R_1 R_2}.\end{aligned} \tag{S20}$$

In the limit $\kappa R \gg 1$, $\delta/R \ll 1$, which is the relevant one for RNA virions, we obtain

$$\mathcal{F}_{\text{elec}} \sim \frac{2\pi\sigma^2 R^2}{\epsilon\kappa}\left(1 - e^{-2\kappa\delta}\right). \tag{S21}$$

It should be noted that Eq. (S21) has a well defined limit for $\kappa \to 0$, which coincides, as it should, with the limit of Eq. (S17) for $\delta/R \ll 1$.

Supplementary Note 3: Scaling theory for an RNA virion at an interface

We can start from Eq. (S21) to build a simple scaling theory for the free energy of an RNA virion at an interface between two media with different values of κ and ϵ, valid for $\kappa R \gg 1$ and $\delta/R \ll 1$. We begin by noting that Eq. (S21) can be written as a contribution proportional to the virion surface embedded in the medium, S, as

$$\mathcal{F}_{\text{elec}} \sim \frac{S\sigma^2}{2\epsilon\kappa}\left(1 - e^{-2\kappa\delta}\right), \tag{S22}$$

which gives an expression for the electrostatic free energy per unit area, $\mathcal{F}_{\text{elec}}/S$. When the virion is at the interface, Fig. 1 in the main text, there are two spherical caps, one in each medium and with surface areas $S_1(z_c) = 2\pi R(R - z_c)$ and $S_2(z_c) = 4\pi R^2 - S_1(z_c)$ for $|z_c| \leq R$, with z_c the interfacial height. We approximate the electrostatic free energy as the sum of the contribution of the two surfaces:

$$\begin{aligned}
\mathcal{F}_{\text{elec}} &\sim \frac{S_1\sigma^2}{2\epsilon_1\kappa_1}\left(1 - e^{-2\kappa_1\delta}\right) + \frac{S_2\sigma^2}{2\epsilon_2\kappa_2}\left(1 - e^{-2\kappa_2\delta}\right) \\
&= \pi\sigma^2 R^2\left(\frac{1 - e^{-2\kappa_1\delta}}{\epsilon_1\kappa_1} + \frac{1 - e^{-2\kappa_2\delta}}{\epsilon_2\kappa_2}\right) + \pi\sigma^2 R z_c\left(\frac{1 - e^{-2\kappa_2\delta}}{\epsilon_2\kappa_2} - \frac{1 - e^{-2\kappa_1\delta}}{\epsilon_1\kappa_1}\right).
\end{aligned} \tag{S23}$$

If we estimate the total free energy of an RNA virion close to an interface as $\mathcal{F}(z_c) = \mathcal{F}_{\text{elec}}(z_c) + \mathcal{F}_{\text{Pick}}(z_c)$, with $\mathcal{F}_{\text{Pick}}$ the Pickering contribution discussed in the main text, and explicitly given by

$$\begin{aligned}
\mathcal{F}_{\text{Pick}}(z_c) &= -\pi\gamma(R^2 - z_c^2), \qquad |z_c| \leq R \\
\mathcal{F}_{\text{Pick}}(z_c) &= 0, \qquad |z_c| \geq R,
\end{aligned} \tag{S24}$$

with γ the surface tension between the two media, then the minimum of $\mathcal{F}$ is obtained with $z_c = z_c^*$, with

$$z_c^* = -\frac{\sigma^2 R}{2\gamma}\left(\frac{1 - e^{-2\kappa_2\delta}}{\epsilon_2\kappa_2} - \frac{1 - e^{-2\kappa_1\delta}}{\epsilon_1\kappa_1}\right). \tag{S25}$$

Consequently, the adsorption free energy is found through the following equation

$$\begin{aligned}
\frac{\Delta\mathcal{F}}{\pi R^2} &= \frac{\mathcal{F}(z_c = z_c^*) - \mathcal{F}(z_c \to -\infty)}{\pi R^2} \\
&= \sigma^2\left(\frac{1 - e^{-2\kappa_2\delta}}{\epsilon_2\kappa_2} - \frac{1 - e^{-2\kappa_1\delta}}{\epsilon_1\kappa_1}\right) - \gamma \\
&\quad - \frac{\sigma^4}{4\gamma}\left(\frac{1 - e^{-2\kappa_2\delta}}{\epsilon_2\kappa_2} - \frac{1 - e^{-2\kappa_1\delta}}{\epsilon_1\kappa_1}\right)^2,
\end{aligned} \tag{S26}$$

Suppl. Fig. 3 shows the phase diagram found for a viral particle close to an interface between two media, found by our scaling theory (see caption for parameter list). The region above the dashed line in Suppl. Fig. 3 corresponds to the adsorbed phase, the one below to the internal phase. The phase diagram is semi-quantitatively similar to the one shown in the main text found by PB numerics, which, unlike this approximate treatment, capture non-linear electrostatics.

Our simulations and theory can be generalised for charge distributions other than the two-shell system modelling an RNA virus. Potentially interesting cases include that of a single charged shell, which models an empty viral capsid, or of a uniform charged sphere, which models the DNA spool inside a bacteriophage. Another relevant case is that where the RNA is not arranged in a uniform layer close to the capsid wall, which corresponds more closely to the case of enveloped viruses [3]. In all these cases, we expect electrostatic interactions at the interface will still be important, although there will be quantitative differences. For instance, in the air phase, the self-energy scales as R^3 for a single charged shell [see Eq. (S8)] and R^5 for a uniform charge distribution, rather than R^2 for the two-shell system representing an RNA virion. While these calculations suggest that these charge distribution will lead to quantitatively different results, such scalings are not directly relevant to our case, as medium III is still likely to be fully aqueous when the virion is at the interface: this will require a modification of our analytical equations.

Supplementary References

[1] A. Šiber and R. Podgornik, Role of electrostatic interactions in the assembly of empty spherical viral capsids, *Phys. Rev. E* **76**, 061906 (2007).

[2] A. Šiber, A. L. Božič, and R. Podgornik, Energies and pressures in viruses: contribution of nonspecific electrostatic interactions, *Phys. Chem. Chem. Phys.* **14**, 3746-3765 (2012).

[3] R. Arranz, R. Coloma, F. J. Chichón,1 J. J. Conesa, J. L. Carrascosa, J. M. Valpuesta, J. Ortín, and J. Martín-Benito, The structure of native influenza virion ribonucleoproteins, *Science* **338**, 1634-1637 (2012).
